# Development and validation of a core-genome multilocus sequence typing scheme for Legionella longbeachae

**DOI:** 10.1099/mgen.0.001467

**Published:** 2025-09-05

**Authors:** Hilary Miller, David Harte, Irina Khokryakova, Ronan Jordan, Amy Bradshaw, Jing Wang, David Winter

**Affiliations:** 1 New Zealand Institute for Public Health and Forensic Science, Porirua, New Zealand

**Keywords:** cgMLST, legionellosis, Legionnaires’ disease, multilocus sequence typing, whole-genome sequencing

## Abstract

*Legionella longbeachae* is a pathogen of global health importance due to its role in causing Legionnaires’ disease (LD), a severe form of community-acquired pneumonia. Throughout the USA and Europe, *Legionella pneumophila* is often identified as the primary cause of LD, but in countries such as New Zealand and Australia, where testing for non-*pneumophila Legionella* species is employed systematically, high rates of *L. longbeachae* are reported. Development of genomic tools to track outbreaks and identify infection sources for *L. longbeachae* has lagged behind that of *L. pneumophila*. This study addresses this deficit through the development of a core-genome multilocus sequence typing (cgMLST) scheme for *L. longbeachae*. Using all 7 publicly available complete genomes and 89 draft genomes, we developed a schema with 2,608 loci at the 95% presence threshold. The schema was validated using 192 isolates, representing both serogroups and all publicly available isolates available as of July 2024. All isolates had 98% or more of the gene targets, indicating the schema is well defined and representative of the breadth of diversity of *L. longbeachae* sequenced to date. Comparison with core-genome SNP analysis showed high concordance between clusters identified with cgMLST and SNP typing, but cgMLST had higher resolution than SNP typing due to the large number of SNPs in recombinant regions that were removed from the analysis. The cgMLST schema will improve the ability of public health laboratories to perform whole genome sequencing-based surveillance of *L. longbeachae* and thus improve our understanding of the global diversity of this pathogen.

## Data Summary

Isolates sequenced in this study have been submitted to the National Center for Biotechnology Information under BioProject accession no. PRJNA1185840. Accession numbers for other samples included in the study are given in Table S1. The cgMLST schema created in this study is publicly available from https://chewbbaca.online/stats.

**Impact Statement**

*Legionella longbeachae* is a common cause of Legionnaires’ disease in New Zealand and Australia and is increasingly being recognized globally as a pathogen of public health importance. Despite this, few genomic tools exist to aid surveillance of this pathogen. In this study, we describe a new core-genome multilocus sequence typing scheme for *L. longbeachae* and show that it is comparable to core-genome SNP typing for identifying clusters, with the advantage of providing standardized results across labs. The schema is publicly available and will greatly improve the ability of public health laboratories to track Legionnaires’ disease outbreaks and investigate transmission sources.

## Introduction

Legionnaires’ disease (LD) is a severe form of community-acquired pneumonia caused by bacteria from the genus *Legionella*, which are ubiquitous in natural aqueous environments and human-associated environments such as cooling towers, hot water distribution systems, spa pools and commercial soil products [1]. Throughout Europe and the USA, LD is primarily caused by *Legionella pneumophila* [2]. However, in New Zealand (NZ), *Legionella longbeachae* is the most common cause of LD, where it was responsible for 55.6% of notified cases between 2010 and 2020 (compared with 23% of cases associated with *L. pneumophila*) [3]. *L. longbeachae* is also prevalent in Australia, and sporadic outbreaks have been reported in Europe [4]. Most cases of * L. longbeachae* in NZ and Australia are associated with commercial compost and potting mixes [5]. *L. longbeachae* may be more prevalent than reported in other parts of the world, as the most commonly used diagnostic test for *Legionella* only identifies * L. pneumophila* serogroup 1. Where PCR and serology testing are employed systematically to test for other *Legionella* species, as is in Australia and New Zealand, higher rates of *L. longbeachae* are generally reported [4].

The development of sequence-based typing tools for *L. longbeachae* has lagged behind that of *L. pneumophila* despite increasing recognition of its importance as a cause of LD. While both multilocus sequence-based typing and core-genome multilocus sequence typing (cgMLST) tools exist for *L. pneumophila* [6,7], no such tools are available for *L. longbeachae*, severely limiting the ability of laboratories to track outbreaks and identify transmission sources.

Core-genome MLST has become a standard typing tool for pathogens now that the use of whole-genome sequencing has become widespread in public health laboratories [8,9]. The schemes used for analysis typically comprise a few thousand loci, enabling greater discriminatory power than classical 7-gene MLST schemes. In addition, the ability to develop systematic allele numbering and cgMLST profile identification enables comparison and standardization of results across different laboratories [10]. Previous genomic studies on *L. longbeachae* have used SNP-based methods to study population structure [11,12]. However, the highly recombinant nature of the *L. longbeachae* genome requires the removal of large numbers of polymorphic sites before SNP typing can be undertaken, thus reducing the resolution of the analysis [12]. Because core-genome MLST is a reference-free, gene-by-gene approach, it reduces the impact of recombination, allowing more of the genome diversity to be included [10].

In this study, we created a cgMLST schema for *L. longbeachae* using all publicly available complete genomes for *L. longbeachae* and 89 draft genomes. We evaluated the scheme on all publicly available genome datasets for *L. longbeachae* that met our quality requirements and compared it to core-genome SNP typing for its ability to identify clusters and isolates known to have the same source.

## Methods

### Bacterial isolates

In New Zealand, legionellosis surveillance activity is undertaken by the Institute for Public Health and Forensic Sciences [PHF Science, formerly the Institute of Environmental Science and Research (ESR), Porirua, New Zealand] on behalf of the NZ Ministry of Health for public health purposes [5]. *Legionella* bacteria are identified from clinical and environmental sources at the PHF Science Legionella Reference Laboratory as part of active source tracing activity for sporadic or outbreak cases of Legionnaires’ disease or from risk assessment activity. Isolates from human clinical samples and soil/compost material sequenced for this study were either referred isolates or isolated at the PHF Science Legionella Reference laboratory from 2018 to 2024. See Table S1 (available in the online Supplementary Material) for the list of isolates.

All isolates were freshly grown on selective GVPC agar (Fort Richard, New Zealand) for 2–3 days at 36 °C before harvesting cells and extracting the genomic DNA using the Geneaid™ MDE480 DNA Extraction Kit (Dnature, New Zealand/Geneaid™, Taiwan), according to the manufacturer’s instructions. The species and serogroup for all isolates were confirmed using a direct fluorescent antibody (DFA) assay (Monoclonal Technologies, Inc., Alpharetta, GA, USA) according to the manufacturer’s instructions and by *mip* gene sequencing [13,14].

### Genomes for the cgMLST schema creation

To construct the cgMLST schema, we used a total of 7 complete genomes and 89 draft genomes (see Table S1). Only seven complete genomes are publicly available for *L. longbeachae*, and these were downloaded from the National Center for Biotechnology Information (NCBI) in October 2023, along with two draft genomes. An additional 65 draft genomes were from PHF Science, and 22 draft genomes were from isolates described in Bacigalupe *et al.* [11]. Draft genomes were chosen based on the availability of high-quality sequence data at the time the schema was created in 2023, and to represent the breadth of diversity in *L. longbeachae* (as determined by serotyping and core-genome SNP analyses).

The PHF Science isolates were sequenced using Illumina paired-end 150 bp sequencing on the NextSeq 550 platform, from libraries prepared using either the Nextera XT (Illumina, San Diego) or Plexwell (Seqwell) library kits. Sequence quality checks, species identification and *de novo* assembly were performed using an in-house pipeline comprising Fastp v. 0.20.1 [15], Centrifuge v.1.0.4 [16], Skesa v. 2.3.0 [17] and Quast v. 5.0.2 [18].

Raw sequence reads for isolates from Bacigalupe *et al.* [11] were downloaded from the NCBI short read archive (SRA), project PRJEB14754. Reads from isolates that were not *L. longbeachae* were excluded. For isolates where two read sets were available, the reads were combined prior to assembly. *De novo* assembly was performed using the Shovill pipeline (https://github.com/tseemann/shovill) with Spades (v.3.15.5) as the assembler. Assembly quality was checked using Quast v. 5.0.2. Depth was calculated by dividing the total number of bases in the reads (calculated by Fastp v. 0.20.1) by 4.1 MB, which is the average genome size for *L. longbeachae*. For the PHF Science and Bacigalupe isolates, only assemblies with depth greater than 40, fewer than 200 contigs and genome size between 4.0 and 4.3 MB were used in the schema creation.

### Creation of the cgMLST schema

The cgMLST schema was created using Chewbbaca v. 3.2.0. A prodigal training file was created using the NSW150 complete genome (Genbank accession no. FN650140). This file was then used to create a whole-genome MLST scheme from the seven complete genomes using the ‘CreateSchema’ operation in Chewbbaca (minimum blast score ratio 0.6). ‘AlleleCall’ was then performed to call the alleles at each locus for the complete genomes, and ‘ExtractCgMLST’ was used to find the set of loci comprising the core genome. This initial cgMLST schema contained 2,696 loci. CgMLST alleles were then called for the 89 draft genomes, and the cgMLST was redetermined using the JoinProfiles and ExtractCgMLST operations.

### Evaluating the schema

CgMLST analyses were performed on 192 *L*. *longbeachae* isolate genomes, including 22 from Bacigalupe *et al*. [11], 52 isolates from Slow *et al*. [12], 13 isolates from the CDC available on NCBI and 105 isolates from the NZ Legionella reference laboratory at PHF Science. This included the 89 isolates used to create the schema. Raw sequence reads for isolates from the CDC and Slow *et al*. [12] were downloaded from the NCBI SRA and *de novo* assembled using the Shovill pipeline (https://github.com/tseemann/shovill) with Spades (v.3.15.5) as the assembler. Assembly quality was checked using Quast v. 5.0.2. Depth was calculated as described above. Only assemblies with depth greater than 40 and fewer than 400 contigs were used in the analysis.

Allele calling was performed using Chewbbaca v. 3.2.0, selecting the scheme with 95% locus-sharing. This scheme retained 2,608 loci. A pairwise cgMLST distance matrix was created from the allele call table produced by Chewbbaca using cgMLST-dists v. 0.4.0 (https://github.com/tseemann/cgMLST-dists), and a neighbour-joining tree based on the cgMLST allelic profiles was created using GrapeTree v. 2.1 [19]. Trees were visualized and annotated using the ggtree package in R v4.3.0.

### Comparison of cgMLST and core-genome SNP typing

Core-genome SNP analyses were performed on the 183 *L*. *longbeachae* isolates previously typed as serogroup 1. A core-genome alignment was built using Snippy v.4.6.0 with the NSW150 genome (Genbank ID: FN650140) as the reference and recombinant sites were removed with Gubbins v. 3.3.5 [20]. The core SNP alignment for the remaining sites was generated with snp-sites v. 2.4.1 [21], and a pairwise SNP distance matrix was produced using snp-dists v. 0.6.3 (https://github.com/tseemann/snp-dists). A maximum likelihood phylogenetic tree was built from the core SNP alignment with IQ-TREE [22] using the built-in model selection and 2,000 bootstrap replicates.

An in-house R script was used to identify clusters from the pairwise distance matrices for both cgMLST and SNP analyses, using thresholds of 15, 25 and 50 for cgMLST allele differences (AD) and 15, 20 and 25 for SNPs. SNP clusters were compared with cgMLST clusters using the adjusted Wallace (AW) coefficient and the adjusted Rand (AR) coefficient [23,24], calculated with an online tool (http://www.comparingpartitions.info/).

## Results and discussion

The cgMLST schema created from seven complete genomes and 89 draft genomes of *Legionella longbeachae* contains 2,608 loci at the 95% loci presence threshold and 2,365 loci at 99% and 100% thresholds. This is smaller than core genomes described by Slow *et al.* [12] (2,952 genes) and Bacigalupe *et al.* [11] (2,574 genes), both of whom calculated the core genome using a pangenome approach. However, both of these studies used only Sg1 isolates of restricted geographic origin (mainly New Zealand isolates in *Slow et al.* [12] and Scottish isolates in Bacigalupe *et al.* [11]). The smaller core genome identified in this study is likely due to the addition of Sg2 isolates and to the wider geographic origin and higher number of isolates used to calculate the core genome. The list of gene targets in the 95% schema and their coordinates is given in Table S2.

The 95% schema was tested on 192 *L*. *longbeachae* isolates representing all available *L. longbeachae* genomes from both the PHF Science and international databases as of July 2024 that were of sufficient depth and quality for analysis. All isolates had 98% or more of the cgMLST loci (mean 99.9% or three loci not found), indicating the schema is well-defined and representative for the breadth of *L. longbeachae* isolates including both serogroups. There were 184 different allele profiles observed among the 192 samples. The mean number of alleles per locus was 3.5 (range 1–21).

**Fig. 1.**
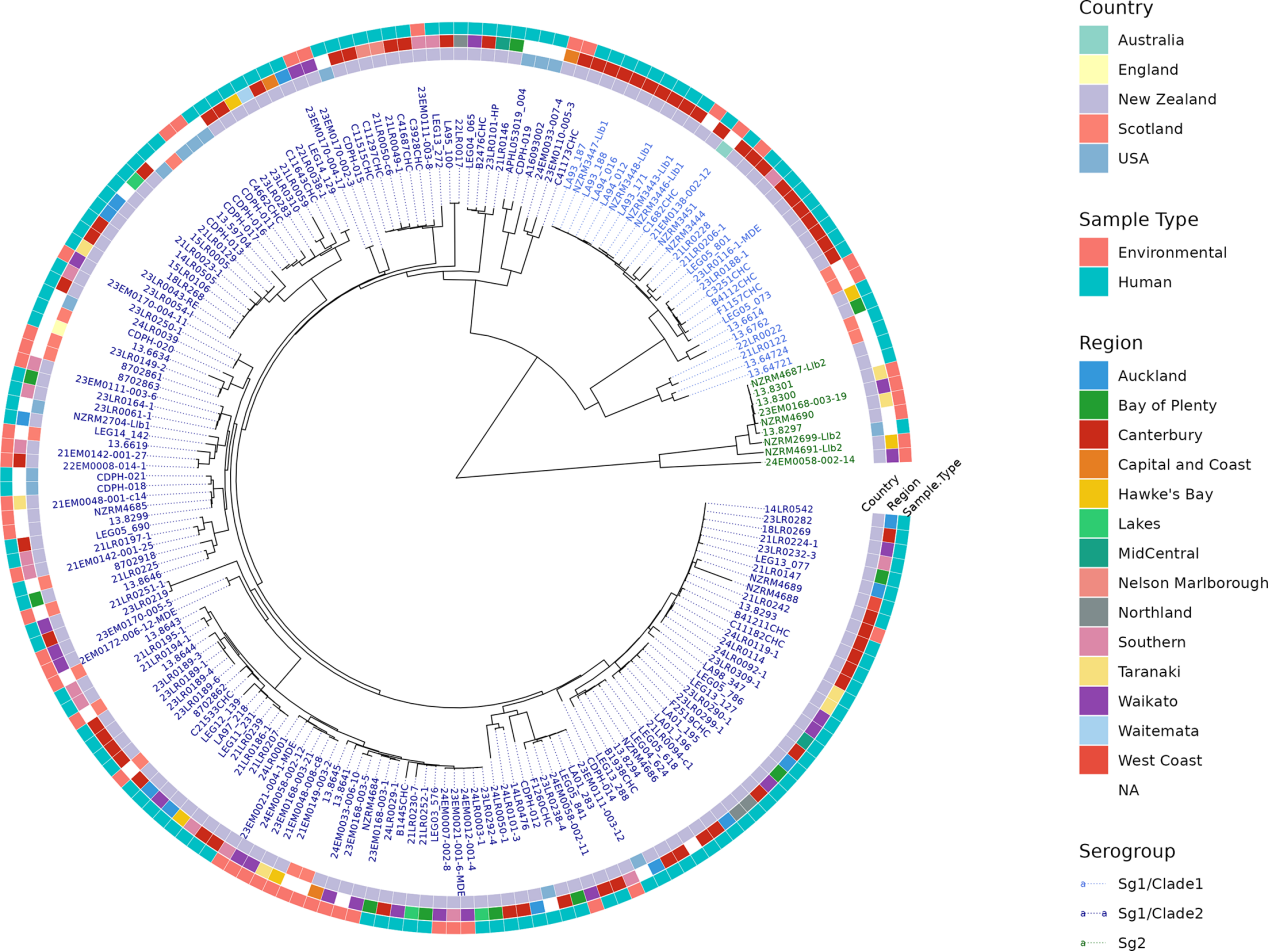
Neighbour-joining distance tree based on cgMLST profiles of 192 *L. longbeachae* isolates from New Zealand, Scotland and the USA. Tree tips are coloured according to serogroup and clade, and the coloured rings around the tree represent (in order from inner to outer rings) country, region (if available) and sample type.

Phylogenetic analysis of cgMLST profiles shows a clear delineation between serogroup 1 and serogroup 2 (Fig. 1). The majority of *L. longbeachae* isolates from all countries belong to serogroup 1, suggesting this serogroup may be more clinically relevant. The tree shows that some lineages appear to be globally distributed, with clades containing isolates from the USA, Scotland and New Zealand and smaller clades of NZ isolates comprising samples from different regions. The two major Sg1 clades identified by Slow *et al*. [12] by core-genome SNP analyses are also resolved by cgMLST (see Sg1/Clade1 and Sg1/Clade2 on Fig. 1). Sg1 clade 1 was identified by Slow *et al*. as being solely comprised of isolates from the Canterbury region in New Zealand and possibly the result of a separate introduction of *L. longbeachae* associated with the import of *Pinus radiata* in the early twentieth century. Our analyses have expanded this clade with further isolates from Canterbury and the Southern region adjacent to Canterbury but also identify isolates from Scotland within the clade that form an immediate outgroup to the Canterbury isolates, as well as an earlier branching subclade of isolates from Scotland and New Zealand. This suggests that the evolutionary history of this clade could be more complex than a single introduction to New Zealand. However, it is difficult to conclude population structure from the limited number of samples available. Although *L. longbeachae* is globally distributed [4], public whole genome sequencing (WGS) datasets are only available for isolates from NZ, Scotland and the USA. The addition of WGS data from countries reporting cases of *L. longbeachae* legionellosis, including Australia, Japan and Europe, would greatly improve our understanding of the geographic structure and spread of this pathogen.

**Fig. 2.**
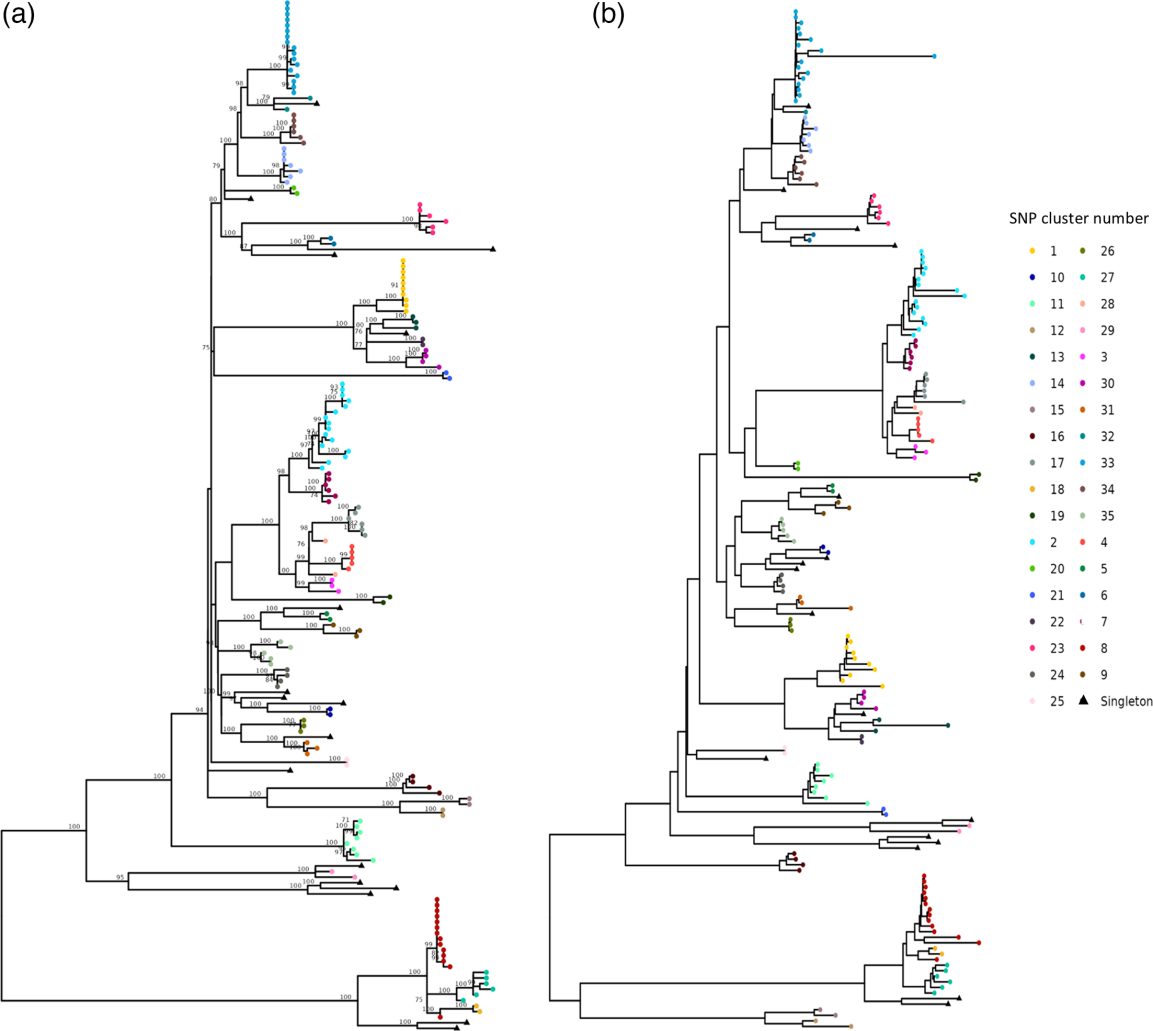
Comparison of core-SNP (a) and cgMLST (b) trees for *L. longbeachae* serogroup 1 isolates. The tree tips are coloured according to the SNP cluster threshold of 20 on both trees. Isolates that do not fall into a cluster are denoted with a black triangle.

A core-genome SNP phylogeny was constructed for Sg1 isolates and compared to the cgMLST data. The Sg1 isolates shared 5,035 core SNPs, but only 1,700 remained after recombination correction with Gubbins. This low number of SNPs not affected by recombination reflects the extent of recombination present in *L. longbeachae*, which is a challenge to phylogenetic reconstruction at longer time scales. The cgMLST scheme may include loci in recombinant regions, and this may affect the determination of ancestral evolutionary relationships, as shown by the difference in branching order of some of the deeper nodes on the cgMLST tree compared with the SNP tree. However, when looking at more recent evolutionary history, there is a high level of concordance between the two trees. Clusters identified on the SNP tree with a threshold of 20 SNPs are also found in the cgMLST tree (shown by the tip colours on (Fig. 2). Comparison of clusters obtained with SNP threshold 15, 20 and 25 and cgMLST AD thresholds 15, 25 and 50 showed highest concordance between clusters for SNP-20/cgmlst-25 (AW=0.879, AR=0.758) and SNP-25/cgmlst-50 comparisons (AW=0.877, AR=0.856) (see Table S3). Note that here we use the term ‘cluster’ to refer to samples that aggregate into a group based on a set AD or SNP threshold, rather than epidemiologically related samples. The cluster thresholds used here were chosen as they are sufficient to discriminate the clades on the tree, but they may not be epidemiologically relevant. An appropriate SNP or cgMLST AD threshold for linking isolates within an outbreak has not been established for *L. longbeachae*, due to a lack of suitable samples for comparison with known epidemiological links. When comparing all isolates to each other, the mean number of cgMLST allele differences between isolates is significantly higher than the number of core SNP differences (cgMLST mean=278.4, SNP mean=113.5, *t*=196.7, *P*<0.001), indicating that cgMLST has higher resolution than SNP typing. This is likely because after correction for recombination, the core SNP genome contains fewer sites than the number of loci in the cgMLST schema (1,700 vs. 2,608).

Our dataset contains several replicate samples, where more than one isolate was sequenced from the same individual or environmental sample (Table 1). The replicate isolates from human clinical cases have almost identical cgMLST profiles (0–1 allele differences), highlighting the suitability of cgMLST in identifying isolates from a common clinical source. However, replicate isolates from the same environmental source (for example, the same bag of potting mix or compost) were generally highly diverse, with up to more than 2,000 allele differences between isolates. Among the samples sequenced at PHF Science for this study, we do not have any examples of linked human and environmental isolates. However, previous studies have found that it is difficult to establish genomic links between human and environmental sources of *L. longbeachae* due to the diversity of *Legionella* present in compost and soil samples [11,25]. This highlights the difficulty with source attribution in Legionellosis outbreaks and shows that the analysis of a large number of isolates using fine-typing methods may be required to find the strain responsible for the clinical infection.

**Table 1.** Range of pairwise cgMLST allele differences among replicate isolates from the same source Each row represents a single source, for example, the same patient for human isolates or the same soil sample for environmental isolates.

	Isolate ID	cgMLST AD	Sample type
1	21LR0194-1, 21LR0195-1	0	Human
2	23LR0189-1, 23LR0189-3, 23LR0189-4, 23LR0189-6	0–1	Human
3	24LR0114, 24LR0119-1	0	Human
4	21EM0142-001-25, 21EM0142-001-27	141	Environmental
5	23EM0111-003-12, 23EM0111-003-6, 23EM0111-003-8	173–246	Environmental
6	23EM0168-003-1, 23EM0168-003-5, 23EM0168-003-19, 23EM0168-003-21	16–2120	Environmental
7	23EM0170-004-11, 23EM0170-004-17	153	Environmental
8	24EM0058-002-11, 24EM0058-002-12, 24EM0058-002-14	235–2114	Environmental

## Conclusion

We have developed a cgMLST scheme comprising 2,608 loci for typing *L. longbeachae*, which is one of the leading causes of Legionnaire’s disease in New Zealand and Australia. The schema is publicly available at https://chewbbaca.online/species/19 and will enable improved tracking of outbreaks and investigation of links to environmental sources. Analysis of all publicly available *L. longbeachae* genomes shows that cgMLST is comparable to core-genome SNP analysis for identifying clusters and samples originating from the same source but has the advantage of inter-lab portability and the potential for setting up a fixed nomenclature scheme for cluster designation. At present, publicly available genomic data for *L. longbeachae* is primarily available from New Zealand and Scotland, and this sampling bias may be reflected in the core-genome scheme developed here. We hope that the availability of this key resource will support further sequencing of *L. longbeachae* from a larger number of countries. Public sharing of such data will improve our understanding of the global population structure and allow this resource to be updated to reflect the global diversity of this pathogen in the future.

## Supplementary material

10.1099/mgen.0.001467 Table S1.

10.1099/mgen.0.001467 Table S2.

10.1099/mgen.0.001467 Table S3.

## Abbreviations

AD: allele differences
cgMLST: core-genome multilocus sequence typing
LD: Legionnaires’ disease
NCBI: National Center for Biotechnology Information
NZ: New Zealand
SNP: single nucleotide polymorphism
WGS: whole genome sequencing
