# Supplementary material for: Development and validation of a core-genome multilocus sequence typing scheme for Legionella longbeachae
Source: Microb Genom. 2025 Sep 5;11(9):001467. doi: 10.1099/mgen.0.001467 (PMC12413299; doi:10.1099/mgen.0.001467)
Supplement: Table S3. [file mgen-11-01467-s003.pdf]

Table S3-A. Adjusted Wallace (AW) values with 95% confidence intervals for clustering at different SNP distance and cgMLST allele difference thresholds. AW measures direction-dependent concordance of clustering methods. It is the probability that two samples are clustered together with method B if they have been clustered together with method A, corrected for chance agreement.

	**snp15**	**snp20**	**snp25**	**cgmlst15**	**cgmlst25**	**cgmlst50**
**snp15**		0.809 (0.654-0.955)	0.748 (0.570-0.914)	0.831 (0.736-0.932)	0.860 (0.756-0.964)	0.677 (0.473-0.866)
**snp20**	0.857 (0.752-0.961)		0.935 (0.851-1.000)	0.703 (0.575-0.834)	0.879 (0.791-0.966)	0.829 (0.688-0.967)
**snp25**	0.793 (0.681-0.904)	0.935 (0.870-1.000)		0.643 (0.511-0.775)	0.819 (0.719-0.917)	0.877 (0.777-0.978)
**cgmlst15**	0.459 (0.290-0.613)	0.366 (0.197-0.516)	0.335 (0.168-0.481)		0.593 (0.416-0.756)	0.293 (0.127-0.437)
**cgmlst25**	0.692 (0.540-0.836)	0.667 (0.481-0.837)	0.621 (0.423-0.801)	0.863 (0.779-0.952)		0.565 (0.355-0.755)
**cgmlst50_**	0.684 (0.529-0.831)	0.790 (0.679-0.897)	0.836 (0.727-0.941)	0.536 (0.384-0.683)	0.710 (0.581-0.832)	

Table S3-B. Adjusted Rand (AR) values with 95% confidence intervals for comparison of SNP and cgmlst clustering at different thresholds. Adjusted Rand estimates the global congruence of two typing methods, corrected for chance agreement.

	**snp15**	**snp20**	**snp25**	**cgmlst15**	**cgmlst25**
**snp20**	0.832 (0.732-0.931)				
**snp25**	0.770 (0.657-0.881)	0.935 (0.879-0.991)			
**cgmlst15**	0.591 (0.439-0.739)	0.482 (0.322-0.633)	0.440 (0.282-0.589)		
**cgmlst25**	0.767 (0.655-0.877)	0.758 (0.628-0.885)	0.706 (0.566-0.842)	0.703 (0.569-0.835)	
**cgmlst50**	0.680 (0.549-0.810)	0.809 (0.717-0.902)	0.856 (0.779-0.934)	0.379 (0.224-0.524)	0.629 (0.487-0.767)
